# Assessment of psychiatric comorbidities and serotonergic or noradrenergic medication use on blood pressure using 24‐hour ambulatory blood pressure monitoring

**DOI:** 10.1111/jch.14311

**Published:** 2021-06-29

**Authors:** Shehzad K. Niazi, Sobia H. Memon, Elizabeth R. Lesser, Emily Brennan, Nabeel Aslam

**Affiliations:** ^1^ Department of Psychiatry & Psychology Mayo Clinic Florida Jacksonville Florida USA; ^2^ Mayo Clinic Robert D. & Patricia E. Kern Center of Science of Health Care Delivery Jacksonville Florida USA; ^3^ Department of Medicine Division of Nephrology & Hypertension Mayo Clinic Florida Jacksonville Florida USA; ^4^ Department of Biostatistics Health Science Research Mayo Clinic Florida Jacksonville Florida USA

**Keywords:** ambulatory blood pressure monitoring, hypertension, psychiatric diagnosis, selective serotonin reuptake inhibitors, serotonin‐norepinephrine reuptake inhibitors

## Abstract

In this study, the authors aimed to assess both nighttime and daytime blood pressure (BP) variability using 24‐hour ambulatory BP monitoring (ABPM) in persons with and without psychiatric conditions and with or without selective serotonin reuptake inhibitors (SSRIs) or serotonin‐norepinephrine reuptake inhibitors (SNRIs) treatment. In this retrospective study, patients who underwent psychiatric evaluation and ABPM within 6 months of each other between January 1, 2012 and December 31, 2017 were identified using billing data. Participants were divided into three groups—participants with no psychiatric diagnosis and no psychiatric medicine (−Diagnosis/−Medication), those with psychiatric diagnosis and on SSRIs/SNRIs (+Diagnosis/+Medication), and psychiatric diagnosis but no psychiatric medications (+Diagnosis/−Medication). Day and nighttime systolic and diastolic BPs were compared between groups controlling for relevant variables using multivariable linear regression models. A total of 475 participants met inclusion criteria including 135 in the −Diagnosis/−Medication group, 232 in the +Diagnosis/+Medication group, and 108 in the +Diagnosis/−Medication group. In adjusted multivariable analysis, the +Diagnosis/+Medication group had higher nighttime systolic BP (median 120 vs 110 mm (Hg); *p* = .01) and nighttime diastolic BP (median 68 vs 63 mm (Hg); *p* = .006) as compared to −Diagnosis/−Medication. No statistically significant differences in BPs between the −Diagnosis/−Medication and +Diagnosis/−Medication groups were observed, after adjustment. Use of SSRIs/SNRIs was associated with significantly higher nocturnal systolic and diastolic BP among patients with psychiatric diagnosis using SSRIs/SNRIs but not associated with psychiatric diagnosis without SSRI/SNRI use. SSRIs/SNRIs use may be associated with higher BP levels and this merits future prospective studies using ABPM to assess day and nighttime BP changes with SSRIs/SNRIs use.

## 1 INTRODUCTION

In 2017, about 7% of all US adults (17.3 million) reported at least one episode of major depression and of those, 11 million reported severe impairment from this episode.^1^, ^2^ Additionally, 19.1% reported having an anxiety disorder in 2017 and 31.1% reported experiencing an anxiety disorder at some point in their life.^3^ Selective serotonin reuptake inhibitors (SSRIs) and serotonin‐norepinephrine reuptake inhibitors (SNRIs) are considered first line treatment choice for most patients with major depression^4^ and various anxiety disorders.^5^ Antidepressant use is common; in 2013, 12% of adults in United States reported using antidepressant medications and eight out of 10 reported long term use.^6^ Furthermore, use of any psychiatric medication was more common among those aged 60–85 years of age (25.1%) compared with those who were 18–39 years old (9.0%).^6^ Association of depression and anxiety with cardiovascular mortality has been previously reported.^7^, ^8^, ^9^, ^10^ This highlights the critical importance of monitoring cardiovascular health and managing any modifiable cardiac and metabolic risk factors in patients with depression and anxiety.

Hypertension (HTN) is an important, common, and potentially modifiable risk factor to consider among those dealing with depression and anxiety. The prevalence of age‐adjusted HTN among adults in United States is 45.4% according to data from national health and nutrition examination survey period 2017–2018.^11^ A number of complications occur due to elevated blood pressure (BP) including cardiovascular disease and chronic kidney disease (CKD).^12^, ^13^ Association of depression by itself and BP has been mixed. It has been linked to hypotension instead of HTN^14^ or no association in a well‐designed population based study.^15^ While anxiety has been positively associated with HTN.^16^ One study has reported that anxiety is associated with nocturnal and early morning HTN.^17^ An elegant hypothesis was put forward decades ago by Friedman and Bennet, who in their study of 1101 participants did not find any association between depression and HTN but did find association between anxiety and HTN^18^: depression causes stress to an individual and this affective‐stress not always but often results in anxiety, which in turn increases BP.^18^

Diagnosis of HTN, its causes, and impact of cardiovascular risk requires repeated BP measurement, clinical assessment, and work‐up including physical examination and laboratory testing.^19^ BP measurement is complicated by the spontaneous variation over the course of the same day and marked reduction occurring during sleep among those with or without HTN.^20^ 24‐hour ambulatory BP monitoring (ABPM) is better able to capture BP changes over the course of the day, than the office‐based BP or home‐based BP measurement. ABPM is a better predictor of changes in response to treatment,^21^ is superior in its reproducibility,^22^ and less prone to phenomenon of “white coat HTN.”^23^ Furthermore, nighttime and daytime HTN both need to be considered. A meta‐analysis of four large prospective European studies found that nighttime BP was a better predictor of mortality and cardiovascular events.^24^ Additionally, other studies have shown the association of nocturnal HTN with adverse cardiovascular outcomes.^25^, ^26^ Nighttime BP is not routinely measured, except when utilizing ABPM.

Antidepressant medications predominantly affect the neurotransmitters serotonin, norepinephrine, and dopamine. Variation in BP might be a result of the effects of these neurotransmitters. Furthermore, there is a positive association between the binding affinities of SSRIs and SNRIs for norepinephrine and serotonin transporters and arterial HTN.^27^, ^28^ Based on this concept, we designed this retrospective study to assess both nighttime and daytime BP variability using 24‐hour ABPM in patients with and without psychiatric conditions and with or without SSRIs/SNRIs treatment. We hypothesized that patients on SSRIs/SNRIs would experience elevated BP, as measured by ABPM.

## 2 MATERIALS AND METHODS

The retrospective study was approved by our institutional review board. All adult patients who had a visit in the department of psychiatry and had an ABPM completed within 6 months of each other between January 1, 2012 and December 31, 2017 at the single study site were identified from billing records. We collected data from the electronic health record on demographics, comorbidities, psychiatric diagnoses, medications, and laboratory results. Patients who were taking psychiatric medicines other than SSRIs/SNRIs were excluded. Detailed ABPM data including daytime and nighttime BP were collected. Spacelab ambulatory blood pressure monitor and software (Spacelabs Healthcare, Snoqualmie, WA, USA) was used for ABPM. BP was evaluated every 10 min during awake hours and every 20 min during the night hours during the 24 hours measurement period. The daytime period was designated as 6:00 a.m.–10:00 p.m. and nighttime was from 10:00 p.m. to 6:00 a.m. The BP cuff bladder covered 80% of the upper arm circumference and placed midline over brachial artery, 2–3 cm above antecubital fossa.

Participants were divided into three groups: participants with no psychiatric diagnosis and no psychiatric medicine (−Diagnosis/−Medication; these patients were potential kidney donors and underwent ABPM and psychiatric evaluation as a part of standard pre‐kidney donation evaluation process), those with a psychiatric diagnosis and on SSRIs/SNRIs (+Diagnosis/+Medication), and those with a psychiatric diagnosis but no psychiatric medications (+Diagnosis/−Medication). Subsequent analyses compared patients in the +Diagnosis/+Medication group who were taking SSRIs to those taking SNRIs.

## 3 STATISTICAL ANALYSIS

All continuous characteristics were summarized with median and first and third quartiles while categorical variables were summarized using frequency and percentage. Differences between groups were compared using the Kruskal–Wallis Rank Sum Test for continuous variables and the Pearson chi‐square test for categorical variables. Single and multivariable linear regression models were used to model subject group differences for ABPM daytime and nighttime BP levels. Possible confounding was accounted for within the multivariable models by adjusting for age, sex, race, history of HTN, history of diabetes mellitus, smoking status, number of antihypertensive medications, and CKD (defined as glomerular filtration rate less than 60 ml/min/1.73 m^2^).^29^ History of diabetes was defined based upon medical record review for diagnosis codes and history of HTN was defined based upon medical record review for diagnosis codes or antihypertensive medications. All tests were two‐sided and performed at the 0.05 significance level. All analysis was performed in the R Statistical Software (version 3.6.2; R foundation for Statistical Computing, Vienna, Austria).

## 4 RESULTS

A total of 475 participants met the inclusion criteria; the −Diagnosis/−Medication group included 135 participants, the +Diagnosis/+Medication group was comprised of 232 participants, and the +Diagnosis/−Medication group consisted of 108 participants. Baseline demographics of the groups are summarized in **Table** **1** and ABPM readings are summarized in **Table** **2**. Overall, the median age of patients was 51 (Q1, Q3: 39, 63) with 61.1% females. Patients in the +Diagnosis/+Medication group were observed to be older with a median age of 57 (Q1, Q3: 43.8, 67.0), have a higher population of females (69.8%), have the highest percentage of White patients (89.2%), and highest percentage of patients with clinically diagnosed HTN (62.5%) and diabetes (21.1%) (*p* < .001). An additional finding regarding patient BP dipping status showed that patients in the +Diagnosis/+Medication group were significantly more likely to be systolic “non‐dippers” (59.7% compared to 38.3% in the −Diagnosis/−Medication group and 44.2% in the +Diagnosis/−Medication group, *p* < .001) (systolic “non‐dipper” defined as a less than 10% decrease in systolic BP in the nighttime compared to daytime BP).^30^

**TABLE 1:** Baseline demographic and comorbid conditions among −Diagnosis/−Medication, +Diagnosis/−Medication, and +Diagnosis/+Medication groups

	−Diagnosis/−Medication (N = 135)	+Diagnosis/+Medication (N = 232)	+Diagnosis/−Medication (N = 108)	Total (N = 475)	*p*‐value
Age	47.0 (36.0, 57.0)	57.0 (43.8, 67.0)	47.0 (34.8, 58.2)	51.0 (39.0, 63.0)	<.001
Female sex	81 (60.0%)	162 (69.8%)	47 (43.5%)	290 (61.1%)	<.001
Race					.005
White	100 (74.1%)	207 (89.2%)	84 (77.8%)	391 (82.3%)	
Black or African American	30 (22.2%)	22 (9.5%)	18 (16.7%)	70 (14.7%)	
Other	3 (2.2%)	2 (0.9%)	5 (4.6%)	10 (2.1%)	
Unknown/not reported	2 (1.5%)	1 (0.4%)	1 (0.9%)	4 (0.8%)	
Diabetes	0 (0.0%)	49 (21.1%)	11 (10.2%)	60 (12.6%)	<.001
Body mass index	27.7 (24.2, 30.4)	28.1 (24.1, 31.9)	26.6 (23.8, 30.2)	27.7 (24.1, 31.1)	.18
Smoking status					<.001
Active	4 (3.0%)	37 (15.9%)	33 (30.6%)	74 (15.6%)	
Former	25 (18.5%)	0 (0.0%)	0 (0.0%)	25 (5.3%)	
Never	106 (78.5%)	195 (84.1%)	75 (69.4%)	376 (79.2%)	
Hypertension‐related characteristics					
History of hypertension	13 (9.6%)	145 (62.5%)	27 (25.0%)	185 (38.9%)	<.001
Glomerular filtration rate					<.001
Less than 60	4 (3.1%)	72 (33.2%)	17 (16.5%)	93 (20.6%)	
60 or higher	127 (96.9%)	145 (66.8%)	86 (83.5%)	358 (79.4%)	
Number of antihypertensives	0.0 (0.0, 0.0)	1.0 (0.5, 3.0)	0.0 (0.0, 0.0)	0.0 (0.0, 2.0)	<.001
Diuretics	1 (0.7%)	69 (29.7%)	10 (9.3%)	80 (16.8%)	<.001
ACE inhibitors or ARBs	11 (8.1%)	83 (35.8%)	13 (12.0%)	107 (22.5%)	<.001
Beta blockers	0 (0.0%)	97 (41.8%)	0 (0.0%)	97 (20.4%)	<.001
Calcium channel blockers	3 (2.2%)	59 (25.4%)	8 (7.4%)	70 (14.7%)	<.001
Other antihypertensives	0 (0.0%)	52 (22.5%)	6 (5.6%)	58 (12.2%)	<.001
Psychiatric disorders					
Adjustment disorder	0 (0.0%)	26 (11.2%)	25 (23.1%)	51 (10.7%)	<.001
Anxiety disorder	0 (0.0%)	134 (57.8%)	22 (20.4%)	156 (32.8%)	<.001
Mood disorder	0 (0.0%)	125 (53.9%)	22 (20.4%)	147 (30.9%)	<.001
Substance‐related disorder	0 (0.0%)	29 (12.5%)	41 (38.0%)	70 (14.7%)	<.001
Personality disorder	0 (0.0%)	3 (1.3%)	0 (0.0%)	3 (0.6%)	.21
Other psychiatric disorder	0 (0.0%)	34 (14.7%)	14 (13.0%)	48 (10.1%)	<.001

Median and first and third quartiles are presented for continuous variables. Frequency and percentages are presented for categorical variables. *p*‐values result from Kruskal–Wallis tests for continuous and Pearson chi‐square tests for categorical variables.

Abbreviations: ACE, angiotensin‐converting enzyme; ARB, angiotensin receptor blockers.

**TABLE 2:** 24‐Hour ambulatory blood pressure result among −Diagnosis/−Medication, +Diagnosis/−Medication, and +Diagnosis/+Medication groups

	−Diagnosis/−Medication (N = 135)	+Diagnosis/+Medication (N = 232)	+Diagnosis/−Medication (N = 108)	*P*‐value
Daytime systolic (mmHg)	124.0 (119.0, 130.0)	131.0 (123.0, 140.0)	126.0 (119.5, 136.5)	<.001
Daytime diastolic (mmHg)	76.0 (72.0, 81.0)	77.0 (71.8, 84.0)	79.0 (72.0, 84.0)	.28
Nighttime systolic (mmHg)	110.0 (104.0, 118.0)	120.0 (110.0, 130.0)	113.0 (103.8, 121.2)	<.001
Nighttime diastolic (mmHg)	63.0 (59.0, 69.0)	68.0 (60.0, 74.0)	66.0 (59.8, 71.2)	<.001

Median and first and third quartiles are presented. *P*‐values result from Kruskal–Wallis tests.

In the unadjusted analysis, participants in the +Diagnosis/+Medication group had higher mean daytime systolic BP than those in the −Diagnosis/−Medication group by an average 7.77 (95% CI: 4.78, 10.76) mm (Hg), higher mean nighttime systolic BP by an average 12.19 (95% CI: 8.79, 15.59) mm (Hg), and higher nighttime mean diastolic BP by an average 4.82 (95% CI: 2.76, 6.88) mm (Hg) (all *p* < .001) (**Table** **3**). After adjustment for age, sex, race, HTN, diabetes, smoking, number of antihypertensive medications, and CKD, participants in the +Diagnosis/+Medication group still had significantly higher nighttime systolic BP compared to the −Diagnosis/−Medication group by 5.45 (95% CI 1.30, 9.59) mm (Hg) (*p* = .01) and nighttime diastolic BP by 3.79 (95% CI: 1.07, 6.51) mm (Hg) (*p* = .006) (**Table** **3**).

**TABLE 3:** Unadjusted and adjusted linear regression analysis for daytime and nighttime blood pressure among −Diagnosis/−Medication, +Diagnosis/−Medication, and +Diagnosis/+Medication groups

	Daytime systolic (mmHg)		Daytime diastolic (mmHg)		Nighttime systolic (mmHg)		Nighttime diastolic (mmHg)	
	Beta (95% CI)	*P*‐value	Beta (95% CI)	*P*‐value	Beta (95% CI)	*P*‐value	Beta (95% CI)	*P*‐value
Unadjusted analysis								
+Diagnosis/+Medication	7.77 (4.78, 10.76)	<.001	1.38 (−0.59, 3.35)	.17	12.19 (8.79, 15.59)	<.001	4.82 (2.76, 6.88)	<.001
+Diagnosis/−Medication	4.28 (0.71, 7.86)	.02	2.27 (−0.08, 4.63)	.06	4.08 (0, 8.15)	.05	2.43 (−0.03, 4.9)	.05

Adjusted analysis								
+Diagnosis/+Medication	0.73 (−2.93, 4.39)	.70	0.83 (−1.84, 3.51)	.54	5.45 (1.3, 9.59)	.01	3.79 (1.07, 6.51)	.006
+Diagnosis/−Medication	1.9 (−1.76, 5.56)	.31	1.13 (−1.55, 3.8)	.41	2.38 (−1.74, 6.5)	.26	1.17 (−1.53, 3.87)	.39

Models adjusted for age, sex, race, history of hypertension, history of diabetes, smoking status, number of antihypertensive medications, and chronic kidney disease.

In order to examine whether higher nighttime systolic and diastolic BP in the +Diagnosis/+Medication group was associated with the psychiatric diagnosis or the use of SSRIs/SNRIs, we compared ABPM results between the −Diagnosis/−Medication group and the +Diagnosis/−Medication group using the same model as for the first analysis. In unadjusted analysis, participants in the +Diagnosis/−Medication group had significantly higher mean daytime systolic BP than the −Diagnosis/−Medication group by 4.28 (95% CI: 0.71, 7.86) mm (Hg) on average (*p* = .02) (**Table** **3**). However, after adjustment, there was no statistically significant difference between these groups both daytime and nighttime systolic or diastolic BP measurements (**Table** **3**).

In order to assess associations of SNRIs and SSRIs with BP separately, we divided the +Diagnosis/+Medication group participants into two subgroups: the SNRI subgroup (participants with psychiatric diagnosis and taking SNRIs) and the SSRI subgroup (participants with psychiatric diagnosis and taking SSRIs). Twelve patients were taking both SSRIs and SNRIs and were excluded from subgroup analysis. The demographics and comorbidities of these subgroups are summarized in **Table** **4** and ABPM readings are summarized in **Table** **5**. No statistically significant differences were observed in unadjusted or adjusted analysis between SNRI and SSRI subgroups for daytime or nighttime systolic and diastolic BP (**Table** **6**).

**TABLE 4:** Baseline demographic and comorbid conditions among SSRI and SNRI groups

	SNRI (N = 66)	SSRI (N = 154)	*P*‐value
Age	56.5 (43.5, 65.0)	57.0 (43.2, 68.0)	.60
Female sex	52 (78.8%)	101 (65.6%)	.05
Race			.62
White	61 (92.4%)	135 (87.7%)	
Black or African American	5 (7.6%)	16 (10.4%)	
Other	0 (0.0%)	2 (1.3%)	
Unknown/not reported	0 (0.0%)	1 (0.6%)	
Diabetes	17 (25.8%)	32 (20.8%)	.42
Body mass index	28.9 (23.9, 33.5)	27.9 (23.6, 30.8)	.19
Smoking status			.04
Active	16 (24.2%)	20 (13.0%)	
Never	50 (75.8%)	134 (87.0%)	
Hypertension‐related characteristics			
History of hypertension	40 (60.6%)	97 (63.0%)	.74
Glomerular filtration rate			.14
Less than 60	16 (25.8%)	52 (36.4%)	
60 or higher	46 (74.2%)	91 (63.6%)	
Number of antihypertensives	1.5 (1.0, 2.0)	1.0 (1.0, 3.0)	.92
Diuretics	18 (27.3%)	49 (31.8%)	.50
ACE inhibitors or ARBs	25 (37.9%)	53 (34.4%)	.62
Beta blockers	23 (34.8%)	70 (45.5%)	.14
Calcium channel blockers	22 (33.3%)	35 (22.7%)	.10
Other misc. antihypertensives	15 (22.7%)	36 (23.5%)	.90
Psychiatric disorders			
Adjustment disorder	7 (10.6%)	18 (11.7%)	.82
Anxiety disorder	42 (63.6%)	84 (54.5%)	.21
Mood disorder	37 (56.1%)	80 (51.9%)	.57
Substance‐related disorder	8 (12.1%)	18 (11.7%)	.93
Personality disorder	2 (3.0%)	1 (0.6%)	.16
Other psychiatric disorder	8 (12.1%)	23 (14.9%)	.58

Median and first and third quartiles are presented for continuous variables. Frequency and percentages are presented for categorical variables. *P*‐values result from Kruskal–Wallis tests for continuous and Pearson chi‐square tests for categorical variables.

Abbreviations: ACE, angiotensin‐converting enzyme; ARB, angiotensin receptor blockers.

**TABLE 5:** 24‐Hour ambulatory blood pressure results among SSRI and SNRI groups

	SNRI (N = 66)	SSRI (N = 154)	*P*‐value
Daytime systolic (mmHg)	130.5 (123.2, 142.0)	131.0 (123.0, 140.0)	.79
Daytime diastolic (mmHg)	78.0 (72.0, 86.5)	76.0 (70.2, 82.8)	.26
Nighttime systolic (mmHg)	120.5 (110.0, 130.0)	119.0 (110.0, 130.2)	.85
Nighttime diastolic (mmHg)	67.5 (60.8, 73.0)	68.0 (60.0, 74.0)	.89

Median and first and third quartiles are presented. *P*‐values result from Kruskal–Wallis tests.

**TABLE 6:** Unadjusted and adjusted linear regression analysis for daytime and nighttime blood pressure among SSRI and SNRI groups

	Daytime systolic (mmHg)		Daytime diastolic (mmHg)		Nighttime systolic (mmHg)		Nighttime diastolic (mmHg)	
	Beta (95% CI)	*P*‐value	Beta (95% CI)	*P*‐value	Beta (95% CI)	*P*‐value	Beta (95% CI)	*P*‐value
Unadjusted analysis								
SSRI	−0.67 (−5.24, 3.9)	.77	−1.85 (−4.87, 1.17)	.23	−0.23 (−5.65, 5.19)	.93	−1.09 (−4.34, 2.17)	.51

Adjusted analysis								
SSRI	−0.65 (−5.11, 3.81)	.78	−1.65 (−4.84, 1.54)	.31	−1.56 (−6.77, 3.64)	.55	−1.81 (−5.19, 1.56)	.29

Models adjusted for age, sex, race, history of hypertension, history of diabetes, smoking status, number of antihypertensive medications, and chronic kidney disease.

## 5 DISCUSSION

Using ABPM, this single‐center retrospective study with 232 patients with psychiatric diagnoses who were using SSRIs/SNRIs demonstrated that these patients had significantly higher nocturnal systolic BP (by an average 5.45 mm Hg) and diastolic BP (by an average 3.79 mm Hg) than patients with no psychiatric diagnoses or SSRIs/SNRIs. This finding may have important clinical implications.

SSRIs and SNRIs, considered first line medications to treat anxiety and depression, can affect BP likely due to effects on serotonin and norepinephrine.^28^ Current literature about the impact of these medications on BP level has shown varying results. Razavi Ratki and colleagues observed in a randomized clinical trial that use of SSRIs induced a greater impact on lowering the diastolic and mean arterial pressure level compared to patients taking other antidepressants.^31^ On the other hand, several studies have suggested that SSRIs, SNRIs, and tricyclic antidepressants might have propensity to increase BP,^32^, ^33^, ^34^, ^35^, ^36^, ^37^ which can potentially increase cardiovascular risk.^38^, ^39^ According to a meta‐analysis of clinical trials, it does appear that SNRIs rather than SSRIs are associated with modest increase in BP.^40^ Our study finding that both SSRIs and SNRIs were associated with significantly higher nocturnal systolic and diastolic BP differs from previously reported findings. This is quite significant because compared with daytime and office BP, nighttime BP is a better predictor of all cause and cardiovascular mortality among both men and women of all ages.^24^

Depression is associated with loss of 28.9 years of quality‐adjusted life expectancy at age 18, among US adults; only 0.41 years of that was due to suicide and 0.26 attributable to depression alone.^41^ Another nationwide study found that anxiety/depression was associated with increased mortality (HR 1.6 with a 95% CI: 1.4, 1.8).^42^ Treating depression and anxiety is important for variety of reasons. Findings of a study conducted by Acharya and colleagues are relevant in this context: it showed a decrease in the mortality rate among patient prescribed SSRIs with concomitant cardiovascular disease.^43^ Additionally, there has also been evidence of beneficial effects of SSRIs use in patients with congestive heart failure and HTN.^44^, ^45^, ^46^, ^47^ Comorbid depression may lead to poorer adherence to antihypertensive treatment leading to polypharmacy,^48^, ^49^ while its treatment can lead to better BP control,^50^ and finally, there is preliminary evidence that integrated treatment of depression and HTN can lead to better outcomes for both.^51^ Thus, the potential risk of increase in nocturnal BP needs to be balanced against the potential beneficial effects of SSRIs/SNRIs use.

Our study methodology of using ABPM has implications for future clinical studies evaluating safety profile of serotonergic and/or noradrenergic psychotropic medications. This has already been proposed for HTN related clinical trials.^22^ Had we not used ABPM, we would have not found the association of SSRIs and SNRIs with higher nocturnal BP. Thus, any study not using ABPM will likely miss important BP changes, especially the nighttime and early morning BP changes. This also poses a clinical practice challenge in terms of routine BP monitoring when prescribing SSRIs/SNRIs especially to those who have pre‐existing HTN. Multiple BP readings throughout the day can potentially approximate the reproducibility and reliability of ABPM^52^ but is cumbersome and not always practical or possible both in clinical and research settings. ABPM is not universally available and especially is not well studied in general psychiatric population presenting in clinical settings as opposed to selected clinical trial populations.

There are several limitations of this study. This was a retrospective study and thus is subject to the impact of other potential confounding variables not measured in this study. Second, though we took into account the presence or absence of psychiatric comorbidities, yet we did not use the severity of each. It is conceivable that severe anxiety or depression has a different level and pattern of impact on daytime and nighttime BP. Additionally, we cannot rule out the that those who had psychiatric diagnoses but were not on psychotropic medications (+Diagnosis/−Medication group) were not using non‐pharmacological treatments such as psychotherapy. It is further possible that study participants in +Diagnosis/−Medication group had less severe psychiatric comorbidities and thus were not receiving treatment. Another limitation of our study is that we used static day (6:00 a.m.–10:00 p.m.) and night (10:00 p.m.–6:00 a.m.) periods to evaluate the BP variation; however, not all individuals maintain the same wake/sleep schedules. The more rigorous approach would be to adjust the software reports to each individual's wake/sleep schedule; however, we were unable to perform this due to limitations of retrospective chart review. Lastly, the retrospective nature of the study also means that even though it found an association between SSRI/SNRI use and nocturnal HTN, yet a causal relationship cannot be established.

Use of a relatively large sample size of a population that is representative of a general psychiatric clinic population where detailed psychiatric evaluations performed by experienced psychiatrists with specialty and sub‐specialty training were available, use of 24‐hour ABPM to assess BP variability, use of a comparison groups −Diagnosis/−Medication (kidney transplant donors) and +Diagnosis/−Medication (psychiatric diagnosis with no SSRIs/SNRIs) are strengths of this study.

## 6 CONCLUSIONS

SSRIs/SNRIs use was associated with a higher nocturnal systolic and diastolic BP. This association needs to be further studied using an appropriately powered longitudinal cohort study using ABPM at the time of psychiatric diagnosis prior to the start of SSRIs/SNRIs and repeating ABPM while receiving treatment. SSRIs/SNRIs, like any other medications, need to be prescribed after careful risk‐benefit analysis, especially in patients with pre‐existing HTN, keeping in mind that sub‐optimal or no treatment of psychiatric comorbidities in itself is associated with considerable risk.

## CONFLICT OF INTEREST

All authors have no conflicts of interest to report.

## AUTHOR CONTRIBUTIONS

Shehzad K. Niazi was responsible for study concept and oversight, interpretation of results, and writing and editing of the manuscript. Sobia H. Memon was responsible for data preparation and maintenance, and writing and editing of the manuscript. Elizabeth R. Lesser was responsible for statistical analysis and writing of the manuscript. Emily Brennan was responsible for statistical analysis and editing of the manuscript. Nabeel Aslam was responsible for study concept and oversight, interpretation of results, and writing and editing of the manuscript.
